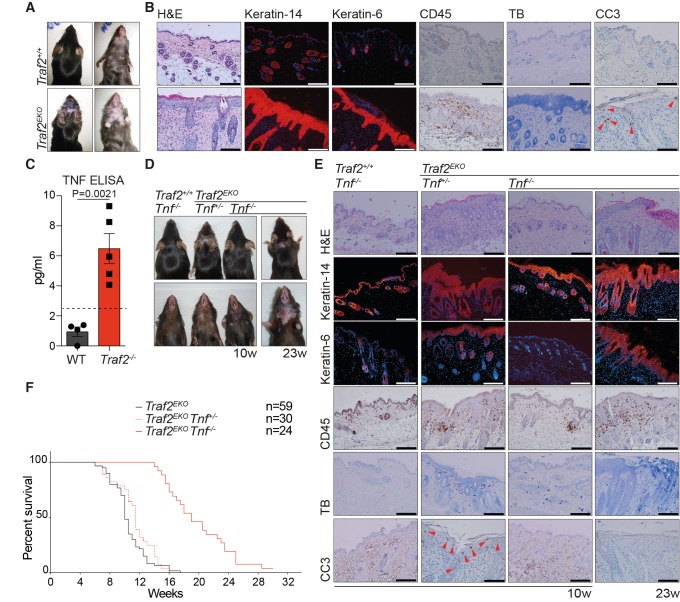# Correction: TRAF2 regulates TNF and NF-κB signalling to suppress apoptosis and skin inflammation independently of Sphingosine kinase 1

**DOI:** 10.7554/eLife.29849

**Published:** 2017-06-27

**Authors:** Nima Etemadi, Michael Chopin, Holly Anderton, Maria C Tanzer, James A Rickard, Waruni Abeysekera, Cathrine Hall, Sukhdeep K Spall, Bing Wang, Yuquan Xiong, Timothy Hla, Stuart M Pitson, Claudine S Bonder, Wendy Wei-Lynn Wong, Matthias Ernst, Gordon K Smyth, David L Vaux, Stephen L Nutt, Ueli Nachbur, John Silke

Etemadi N, Chopin M, Anderton H, Tanzer MC, Rickard JA, Abeysekera W, Hall C, Spall SK, Wang B, Xiong Y, Hla T, Pitson SM, Bonder CS, Wong WW-L, Ernst M, Smyth GK, Vaux DL, Nutt SL, Nachbur U, Silke J. 2015. TRAF2 regulates TNF and NF-κB signalling to suppress apoptosis and skin inflammation independently of Sphingosine kinase 1. *eLife* **4**:e10592. doi: 10.7554/eLife.10592.

Published 23, December 2015

We were recently alerted to two errors in our recent eLife publication. Firstly, the anti JNK panel in Figure 2B contained 11 lanes when it should, like all the other blots in this panel, have had 12 lanes. In the original experiment we also ran four lanes of lysates from *Sphk2*^-/-^ cells treated with TNF. We did not include these in the final paper because they behaved like *Sphk1*^-/-^ and wild type cells and were superfluous. Unfortunately when cropping the images to remove this extraneous data we accidently removed one of the *Sphk1*^-/-^ lanes in the anti-JNK blot. Upon carefully re-examining this data we also discovered that the protein marker for this panel was incorrectly recorded as 45kD rather than 49kD. These errors have now been corrected. Secondly, three Toluidine Blue (TB) stained panels in Figure 4B and 4E are identical. This duplication occurred unintentionally during the assembly of the figures using Adobe Illustrator. Figure 4 has now been corrected with the appropriate images. In generating this response we provided full scans and primary data to the editors of eLife and would likewise be happy to share this information with any interested reader. We apologise for these mistakes, for any inconvenience they may have caused, and would like to thank the reader for bringing them to our attention.

Original Figure 2:

**Figure 1:**
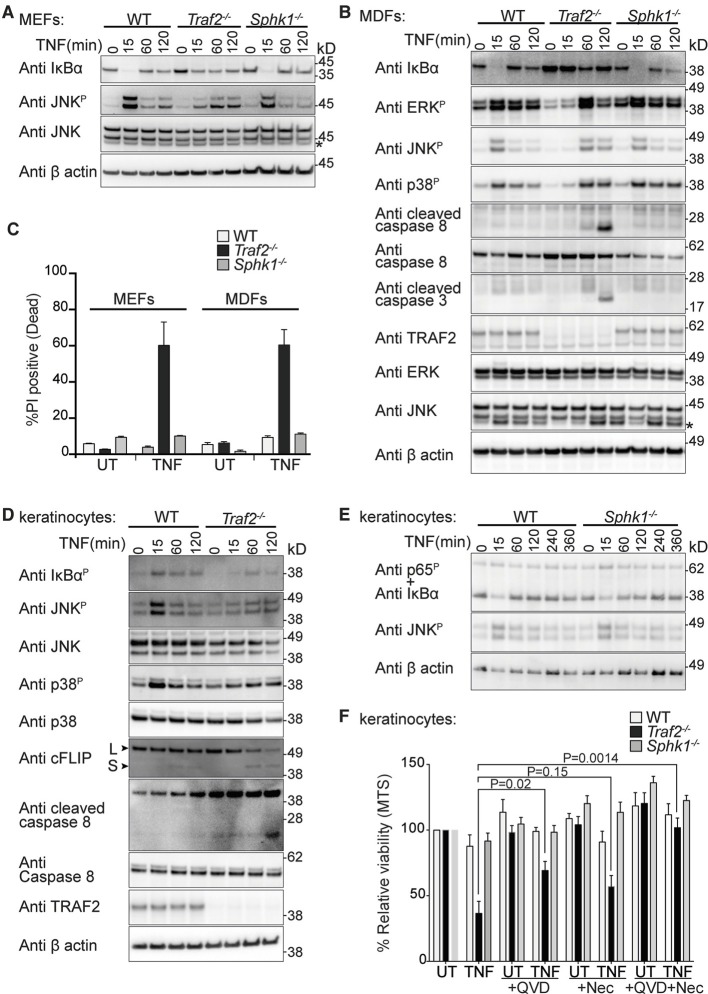

Corrected Figure 2:

**Figure 2:**
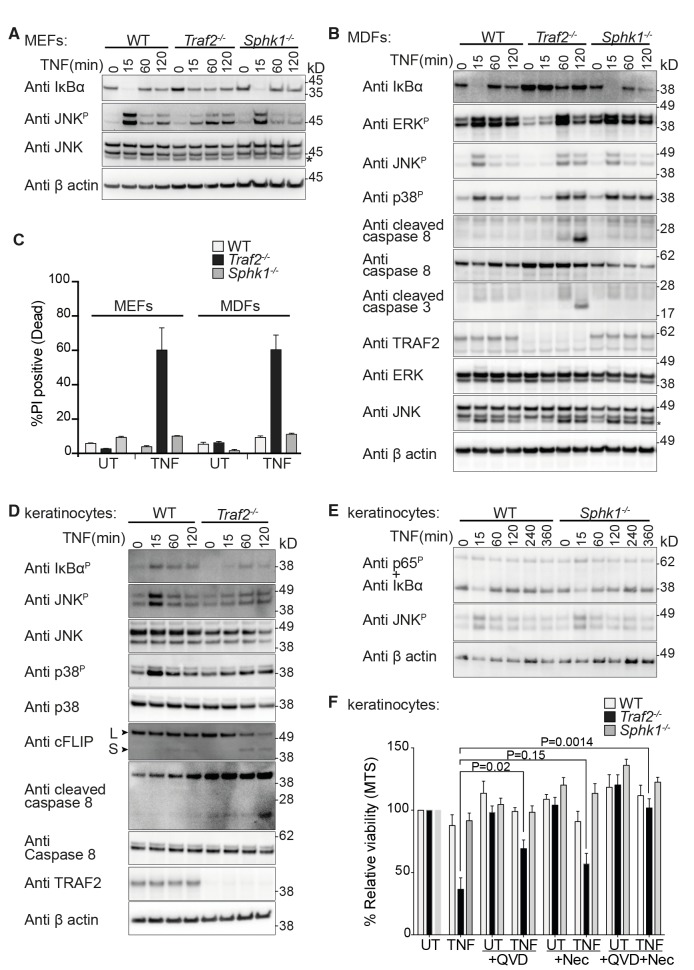

Original Figure 4:

**Figure 3:**
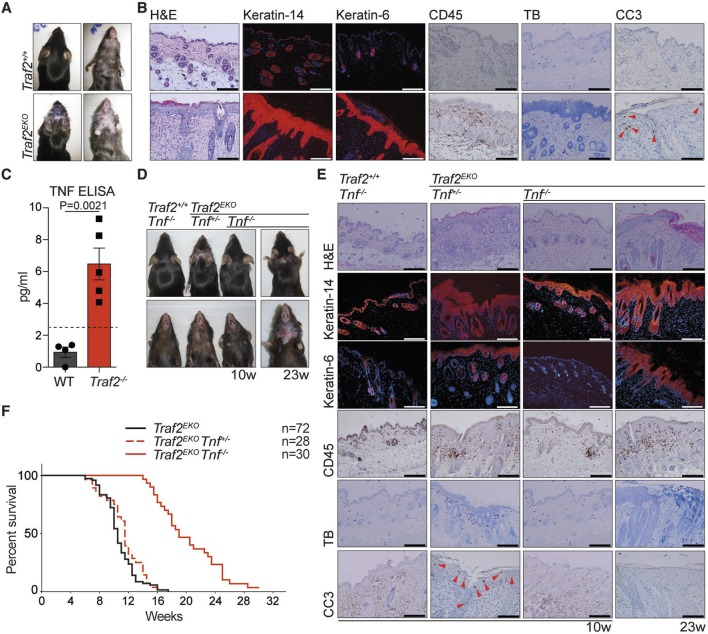

Corrected Figure 4:

**Figure 4:**