# Supplementary figures and images for: TRAF2 regulates TNF and NF-κB signalling to suppress apoptosis and skin inflammation independently of Sphingosine kinase 1
Source: eLife. 2015 Dec 23;4:e10592. doi: 10.7554/eLife.10592 (PMC4769158; doi:10.7554/eLife.10592)

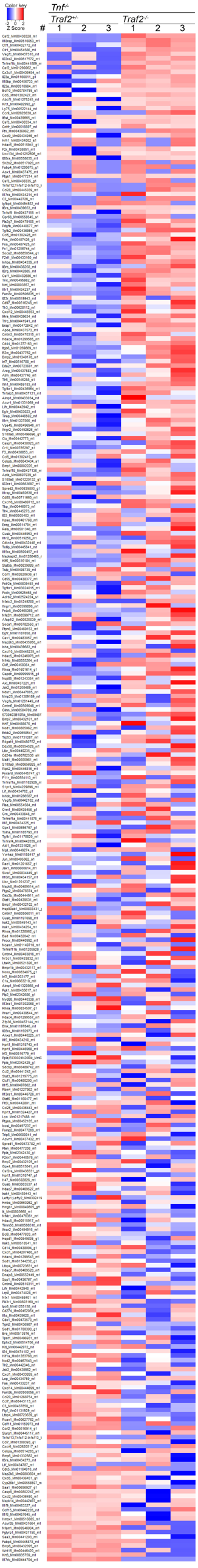

Supplement: Figure 8—source data 1. — Heat map depicting a selection from qPCR analysis of more than 600 inflammatory genes from Tnf-/-Traf2+/- and Tnf-/-Traf2-/- keratinocytes (3 mice for each genotype). Log expression values have been standardized to have mean 0 and standard deviation 1 for each row. Genes were ranked based on the fold change expression. DOI: http://dx.doi.org/10.7554/eLife.10592.011 [file elife-10592-fig8-data1.zip › Etemadi et al_ Source data file 1.jpg]
